# Flagellar Basal Body Structural Proteins FlhB, FliM, and FliY Are Required for Flagellar-Associated Protein Expression in *Listeria monocytogenes*

**DOI:** 10.3389/fmicb.2018.00208

**Published:** 2018-02-13

**Authors:** Changyong Cheng, Hang Wang, Tiantian Ma, Xiao Han, Yongchun Yang, Jing Sun, Zhongwei Chen, Huifei Yu, Yi Hang, Fengdan Liu, Weihuan Fang, Lingli Jiang, Chang Cai, Houhui Song

**Affiliations:** ^1^China-Australian Joint Laboratory for Animal Health Big Data Analytics, Zhejiang Provincial Engineering Laboratory for Animal Health Inspection and Internet Technology, College of Animal Science and Technology, Zhejiang A&F University, Lin’an, China; ^2^Zhejiang University Institute of Preventive Veterinary Medicine and Zhejiang Provincial Key Laboratory of Preventive Veterinary Medicine, Hangzhou, China; ^3^Department of Health Services and Management, Ningbo College of Health Sciences, Ningbo, China; ^4^School of Veterinary and Life Sciences, Murdoch University, Perth, WA, Australia

**Keywords:** flagella, *Listeria monocytogenes*, motility, protein expression, type III secretion system

## Abstract

*Listeria monocytogenes* is a food-associated bacterium that is responsible for food-related illnesses worldwide. In the *L. monocytogenes* EGD-e genome, FlhB, FliM, and FliY *(encoded* by *lmo0679, lmo0699*, and *lmo0700*, respectively) are annotated as putative flagella biosynthesis factors, but their functions remain unknown. To explore whether FlhB, FliM, and FliY are involved in *Listeria* flagella synthesis, we constructed *flhB, fliM, fliY*, and other flagellar-related gene deletion mutants using a homologous recombination strategy. Then, we analyzed the motility, flagella synthesis, and protein expression of these mutant strains. Motility and flagella synthesis were completely abolished in the absence of *flhB, fliM*, or *fliY*. These impaired phenotypes were fully restored in the complemented strains CΔ*flhB*, CΔ*fliM*, and CΔ*fliY*. The transcriptional levels of flagellar-related genes, including *flaA, fliM, fliY, lmo0695, lmo0698, fliI*, and *fliS*, were downregulated markedly in the absence of *flhB, fliM*, or *fliY*. Deletion of *flhB* resulted in the complete abolishment of FlaA expression, while it decreased FliM and FliY expression. The expression of FlaA was abolished completely in the absence of *fliM* or *fliY*. No significant changes were found in the expression of FlhF and two flagella synthesis regulatory factors, MogR and GmaR. We demonstrate for the first time that FlhB, FliM, and FliY not only mediate *Listeria* motility, but also are involved in regulating flagella synthesis. This study provides novel insights that increase our understanding of the roles played by FlhB, FliM, and FliY in the flagellar type III secretion system in *L. monocytogenes*.

## Introduction

The gram-positive bacterium *Listeria monocytogenes* is a ubiquitous intracellular pathogen, which has been implicated within the past decades as the causative organism in several outbreaks of foodborne disease. The bacterial flagellum is an ultra-large molecular complex composed of over 30 protein components (Morimoto and Minamino, 2014). In Gram-positive and Gram-negative bacteria, the flagellum is composed of three major parts, including the flagellar filament, the hook complex, and the basal body, which crosses the bacterial cell membrane, as well as a flagellar-associated cytoplasmic ring. Flagellar-related proteins are transported through the basal body to the outside of the cell, where they are assembled into the helical hook and filament complexes (Berg, 2003). Bacterial swimming is controlled by flagella in accordance with alternations of environment, through clockwise (CW) or counterclockwise (CCW) rotation of motors (Blair, 2003). For example, in *Escherichia coli* CCW rotation allows the several filaments to join in a bundle on a cell and drive it smoothly forward (a ‘run’), while CW rotation destroys the filament bundle and causes rapid somersaulting (a ‘tumble’) (Blair, 2003), while binding of the response regulator phosphorylated CheY (CheY-P) to the rotor switch component FliM and FliN will change the direction of motor swimming (Delalez et al., 2010). FliG, FliM, and FliN belong to the cytoplasmic ring structure, while the MotA/MotB complex is a stator of the flagellar motor that acts as a H^+^ channel to couple the proton flow with torque generation (Hosking and Manson, 2008; Onoue et al., 2016). In the process of the bacterial flagella biosynthesis, the greatest challenge is the coordination of the complex flagellar assemble, which requires the timely transcription of the flagellar-associated genes, fine-tuning of intracellular processing, folding and export of flagellar proteins, and the final formation of a functional flagellar filament (Liu and Ochman, 2007). There are dozens of flagellar-related proteins in *L. monocytogenes*, which serve as regulators, structural proteins, cofactors, an ATPase, and proteins that are required protein transport and modification. These proteins play an important role in flagellar synthesis. Flagellar synthesis in *L. monocytogenes* is controlled mainly by the interaction of MogR and GmaR. The process of *Listeria* flagella biosynthesis is temperature dependent and finely regulated through an obviously different mechanism than the well-described hierarchical regulation of gram-negative bacteria (Grundling et al., 2004). Thus, at mammalian host physiologic temperature, 37°C, most *L. monocytogenes* strains do not produce flagella and are non-motile (Peel et al., 1988; Shen and Higgins, 2006). This is due to MogR repression of flagellar gene transcription at 37°C (Grundling et al., 2004). In contrast, at 30°C and below, *L. monocytogenes* is motile because MogR is inhibited by its anti-repressor GmaR, thus permitting flagellar gene transcription (Shen et al., 2006). DegU is also involved in regulating flagellar-related gene transcription, which in turn affects flagellar synthesis and bacterial motility (Williams et al., 2005). The flagellar-mediated transport and secretion system in most Gram-negative pathogenic bacteria is a type III secretion system (T3SS). The flagellar T3SS is closely related to the effector toxin-exporting T3SSs of pathogenic bacteria. The pathogen-associated T3SSs are similar to the majority of the flagellar T3SSs, but the specific protein transport mechanism is not yet clear. FlhB, FliM, and FliY are involved mainly in the formation of the flagellum in Gram-negative bacteria, and they are flagellar T3SS component proteins that mediate the transport and of flagellar-related proteins (Liao et al., 2009; Meshcheryakov et al., 2013).

Among various bacteria, the pathogenic non-flagellar T3SS is merely found in Gram-negative bacteria, and in Gram-positive bacteria has not been reported. The bacterial flagellar-T3SS and the non-flagellar T3SS have a high degree of homology, both of the two systems can secrete virulence proteins, so there is the structural distinction as well functional similarity (Duan et al., 2013). For example, in Gram-positive *Bacillus thuringiensis*, secretion of some virulence determinants is dependent on a functional flagellar export apparatus (Young et al., 1999; Ghelardi et al., 2002). In Gram-negative *Campylobacter jejuni*, due to the lack of a classical T3SS, the flagellar export system therefore plays a major role in secreting the virulence factors during bacterial infection (Konkel et al., 2004).

The function of the FlhB, FliM, and FliY proteins in *L. monocytogenes* has not been reported. To better understand the relationship between flagellar gene expression and assembly of the flagella in *L. monocytogenes*, genes encoding various export apparatus components were disrupted, and bacterial motility, flagellar biosynthesis, and the transcript levels of the selected flagellar genes were studied. First, we used genetic recombination techniques to construct deletion mutants of *flhB, fliM*, and *fliY*. Second, the phenotypes, including motility, flagellar assembly, growth, flagellar-related gene transcription, and changes in flagellar protein levels, of the deletion mutants were analyzed. The results showed that FlhB, FliM, and FliY are involved in flagellar synthesis and motility, as well as the expression, transport of flagellar-related proteins. Our results show, for the first time, that FlhB, FliM, and FliY are required for flagella synthesis and motility in *L. monocytogene*. These results lay the foundation for further exploration of the flagellar T3SS in *L. monocytogenes*.

## Results

### The Flagellar Genes *flhB, fliM*, and *fliY* Are Not Required for Growth *in Vitro*

**FIGURE 1:**
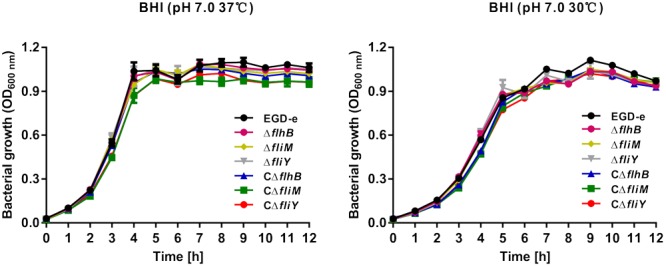
Growth of *L. monocytogenes* mutants in BHI medium at 37°C **(A)** and 30°C **(B)**. Overnight cultures were resuspended in fresh BHI medium and then incubated at 37°C **(A)** and 30°C **(B)** for 12 h. Then, the OD_600 nm_ was measured at 1-h intervals. The data are expressed as the mean ± standard deviation (*SD*) of three independent experiments.

The *flhB, fliM*, and *fliY* deletion mutants were constructed successfully, and then their phenotypes were analyzed. The mutant strains were grown at different temperatures and there were virtually no significant growth differences among the strains at 30°C and 37°C, compared with the wild-type strain and the complemented strains CΔ*flhB*, CΔ*fliM*, and CΔ*fliY* (**Figures 1A,B**), suggesting that *flhB, fliM*, and *fliY* are not essential for growth *in vitro*.

### The *L. monocytogenes* Mutant Strains Δ*flhB*, Δ*fliM*, and Δ*fliY* Lack Flagellar and Are Non-motile

**FIGURE 2:**
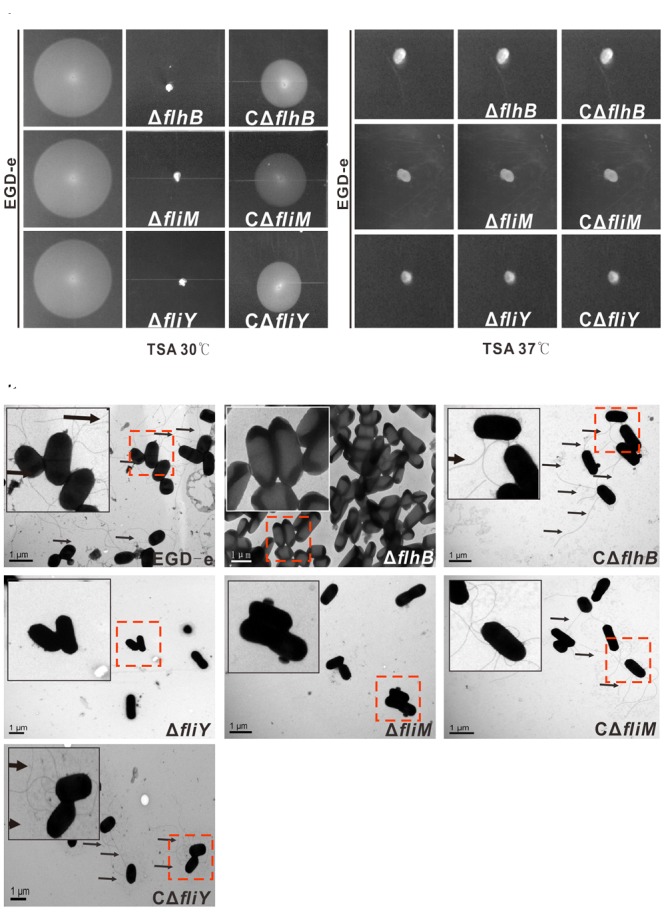
FlhB, FliM, and FliY contribute to *L. monocytogenes* motility and flagellar formation. A motility assay **(A,B)** and TEM **(C)** were performed by growing the *L. monocytogenes* wild-type strain EGD-e, the mutants Δ*flhB*, Δ*fliM* and Δ*fliY*, and the complemented strains CΔ*flhB*, CΔ*fliM*, and CΔ*fliY* on soft agar (0.25%) at 30°C or 37°C for 16 h. Scale: 1 μm.

The motility of the *flhB, fliM*, and *fliY* mutant strains was abolished completely in semisolid culture medium (tryptic soy agar) at 30°C, while the wild-type strain (EGD-e) and the complemented strains produced circular “swarms” (**Figure 2A**). All the strains were non-motile at 37°C, which inhibited flagellar formation in many *Listeria* strains (**Figure 2B**) (Grundling et al., 2004). Therefore, it can be seen that FlhB, FliM, and FliY are critical for the motility of *L. monocytogenes*, as any single mutant abolished motility completely. Because of the loss of motility in the mutants, we wondered whether the mutants adversely affected flagellar synthesis. Therefore, we used transmission electron microscopy (TEM) to examine whether these mutants produced flagella. The results showed that the Δ*flhB*, Δ*fliM*, Δ*fliY* mutants did not produce any flagellar filaments, while the EGD-e, CΔ*flhB*, CΔ*fliM*, and CΔ*fliY* strains did (**Figure 2C**). These results clearly show that the loss of *flhB, fliM*, and *fliY* abolished flagellar synthesis and motility in *L. monocytogenes*.

### Changes in the Levels of Flagellar-Associated Gene Transcripts in the Δ*flhB*, Δ*fliM*, and Δ*fliY* Strains

**FIGURE 3:**
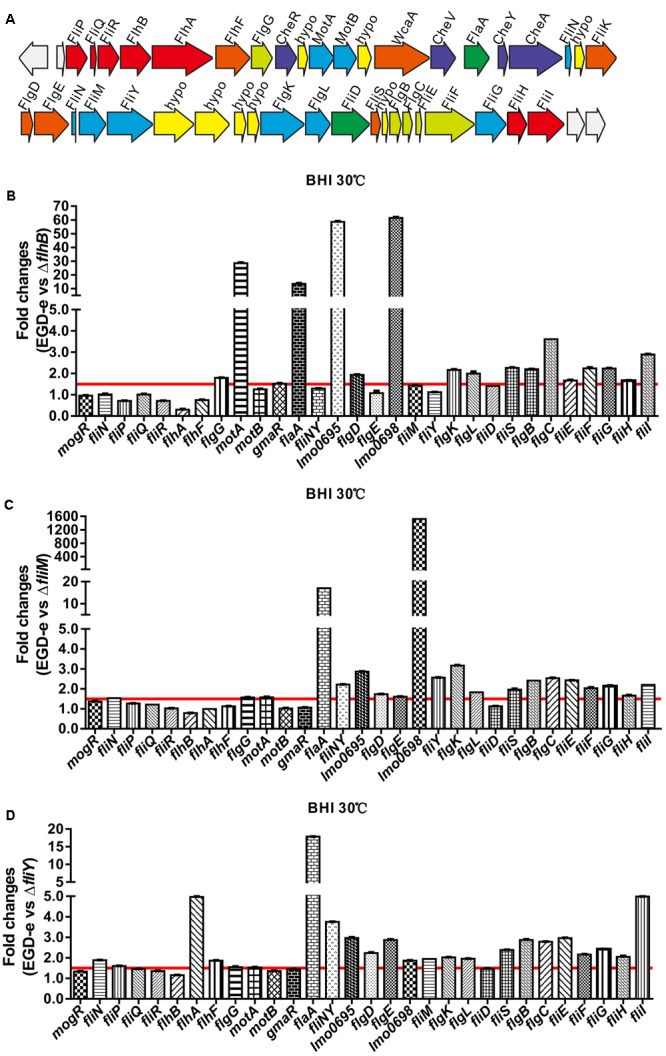
The flagellar-motility gene cluster in *L. monocytogenes* EGD-e **(A)** and the changes of the transcriptional levels of flagellar-related genes in the absence of *flhB* **(B)**, *fliM* **(C)**, or *fliY* **(D)**. Relative quantification of flagellar-related gene mRNA levels in the *L. monocytogenes* wild-type strain EGD-e, the mutants Δ*flhB*, Δ*fliM*, and Δ*fliY*, and the complemented strains CΔ*flhB*, CΔ*fliM*, and CΔ*fliY* at 30°C. Values are expressed as the mean ± SD. Solid lines indicate a 1.5-fold change in transcription of the genes of interest.

Genes encoding the flagellar-associated genes from *L. monocytogenes* EGD-e are present in a 41-gene cluster encoding proteins related to flagellar and motility (**Figure 3A**) (Desvaux and Hebraud, 2006). We used a quantitative real-time PCR (qRT-PCR) to demonstrate that the deletion of *flhB* resulted in the downregulation of the transcription of most flagellar-associated genes (**Figure 3B**, the ratio of expression of the wild-type strain EGD-e to that of the *ΔflhB* strain was ≥1.5), and the transcription of *flaA, fliY, fliS, motA, lmo0695, lmo0698* was downregulated significantly (**Figure 3B**). The absence of *fliM* also resulted in the downregulation of the transcription of most flagellar-associated genes (**Figure 3C**, the ratio of expression of the wild-type strain EGD-e to that of the Δ*fliM* strain was ≥1.5), and *flaA, lmo0698, lmo0695, flgK, fliS*, and *fliI* transcription was downregulated significantly (**Figure 3C**). Similarly, the deletion of *fliY* resulted in the downregulation of the transcription of most flagellar-associated genes (**Figure 3D**, the ratio of expression of the wild-type strain EGD-e to that of the Δ*fliY* strain was ≥1.5), and the transcription of *flaA, flhA, fliI*, and *fliNY* was downregulated significantly (**Figure 3D**). In these three mutants, the transcription of genes in the *fliN, flaA, lmo0696*, and *lmo0703* operons was mainly downregulated. The results confirm that *flhB, fliM*, and *fliY* are essential for the transcriptional regulation and synthesis of other genes related to bacterial flagella.

### FlhB, FliM, and FliY Are Involved in the Expression of Flagellar-Associated Proteins

**FIGURE 4:**
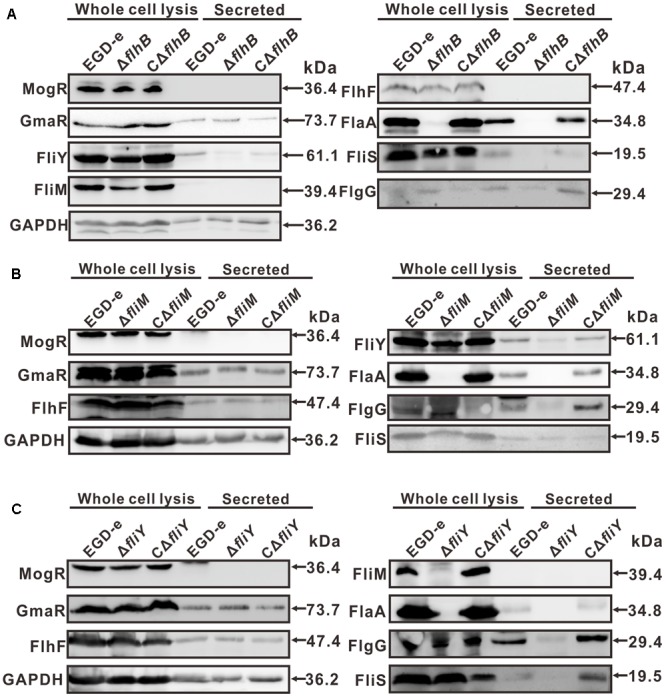
Changes in the levels of flagellar-related proteins in the absence of *flhB* **(A)**, *fliM* **(B)**, or *fliY* **(C)**. Bacterial overnight cultures of the *L. monocytogenes* wild-type strain EGD-e, the Δ*flhB*, Δ*fliM*, Δ*fliY* mutants, and the complemented strains CΔ*flhB*, CΔ*fliM*, and CΔ*fliY* were diluted 1:100 into 100 ml of fresh BHI broth and statically grown to stationary phase. Bacterial sediments and culture supernatants were collected to obtain the different cell fractions. Proteins were separated by 12% SDS-PAGE and immunoblotted with α-FlgG, α-MogR, α-FlhF, α-FliM, α-FliY, α-FlaA, α-FliS, or α-GmaR antisera. GAPDH was used as an internal control. The predicted molecular weight of each protein is indicated on the right.

In the Δ*flhB* strain, the expression of FlaA was abolished completely, while FliS was expressed. Moreover, the expression of the cytoplasmic proteins FliY and FliM was reduced fractionally, while the expression FlhF and the regulatory factors MogR and GmaR was not affected (**Figure 4A**). The results indicate that FlhB can adversely affect the expression of some flagellar-associated proteins. The absence of *fliM* resulted in the complete loss of expression of the secretory of FlaA. Additionally, the expression of the cytoplasmic protein FliY was attenuated slightly as well, while the expression of FlhF and the transcriptional regulators MogR and GmaR was not affected (**Figure 4B**). The results show that FliM adversely affects the expression and secretion of bacterial flagellar-associated proteins. Furthermore, the results showed that the deletion of *fliY* resulted in the complete loss of expression of FliM and FlaA, the expression of FlhF and the transcriptional regulatory factors MogR and GmaR was not affected (**Figure 4C**). These results demonstrate that FliY adversely affects the expression and of flagellar-associated proteins. Collectively, these results are consistent with the previous motility assay, the results of the TEM analysis, and the aforementioned changes of the transcript levels of flagellar genes.

## Discussion

The bacterial flagellum has previously been shown to play a critical role as an export apparatus in mediating extracellular secretion of non-flagella virulence factors and other important heterologous polypeptides (Haiko and Westerlund-Wikstrom, 2013). The effect of flagella on bacterial pathogenicity in Gram-positive bacteria has not been reported, but the effect of flagella on the pathogenicity of Gram-negative bacteria is important (Osterman et al., 2015). Flagellar not only mediate the movement of bacteria, but they are also involved in bacterial pathogenicity through the flagellar T3SS secretion system, which is mainly involved in effector transport (Bange et al., 2010; Osterman et al., 2015).

In Gram-negative bacteria, FlhB is a constituent protein of the basal body (Casey et al., 2014; Diepold and Armitage, 2015), and FlhB is encoded by *lmo0679* in *L. monocytogenes* strain EGD-e. In this study, the absence of *flhB* resulted in the inability to synthesize flagellar (**Figure 2C**), which resulted in a non-motile phenotype on tryptic soy agar semi-solid medium (**Figure 2**). This indicates that the Δ*flhB* mutant cannot form flagellar and that it is non-motile at a low temperature. The qRT-PCR results showed that the deletion of *flhB* led to the downregulation of the transcription of most flagellar-related genes (**Figure 3B**). The highly conserved membrane protein FlhB, as an important component of the flagellar secretion system, plays an active role in regulating proteins export (Meshcheryakov et al., 2013). The analysis of expression of flagellar-related proteins in the deletion mutants showed that in the Δ*flhB* mutant, the expression of FlaA was abolished completely. Additionally, the expression of the cytoplasmic proteins FliY and FliM was reduced slightly, while the expression of FlhF and the regulatory factors MogR and GmaR was not affected (**Figure 4B**). These results indicate that FlhB affects the expression and secretion of flagellar-associated proteins. This study, for the first time, showed that FlhB is involved in regulating the flagellar synthesis of important flagellar proteins, and that it affects the secretion of other bacterial proteins in *L. monocytogenes*. The main function of FlhB in *Yersinia pseudotuberculosis* is to mediate the transport of T3SS effector molecules and mediate the transport of bacterial toxins into host cells (Minamino and Macnab, 2000a; Macnab, 2004; Barker and Samatey, 2012; Login and Wolf-Watz, 2015; McMurry et al., 2015).

Flagellar-associated proteins in the majority of Gram-negative bacteria and only a few in Gram-positive bacteria are found involved in the synthesis and transport of effector molecules (Ghelardi et al., 2002; Diepold and Armitage, 2015). The main components of the flagellar T3SS are located mainly in the cytoplasmic membrane of bacteria, and they include FlhA, FlhB, FliP, FliQ, FliR, and FliO, which constitute the intramembrane protein complex (Minamino and Macnab, 1999). The function of the flagellar T3SS is to transport flagellar-related proteins out of the cell. Cytoplasmic flagellar proteins include FliI, FliH, and FliJ. FliI is ATPase (Fan and Macnab, 1996); FliH is a negative regulator of FliI (Minamino and MacNab, 2000b), whereas FliJ is a molecular chaperone that facilitates the transport of flagellar T3SS proteins (Minamino et al., 2000; Stafford et al., 2007). The key protein in the cytoplasmic membrane complex is FlhA, and the C-terminal domains of both FlhA and FlhB are predictively located in the cytoplasm and might be involved in substrate translocation and substrate specificity switching, respectively (Minamino and MacNab, 2000c). In *Bacillus thuringiensis*, FlhA mediates the transport of bacterial virulence factors into host cells, which is required for pathogenesis (Ghelardi et al., 2002), but its mechanism of action has not been demonstrated. The function of FlhB has not been reported in Gram-positive bacteria except *B. subtilis* (Calvo and Kearns, 2015). However, in Gram-negative bacteria and some thermophiles, FlhB and the hook-length control protein FliK form an export switching machinery that switches export specificity of the flagellar-T3SS upon completion of the hook structure, thereby coordinating flagellar gene expression with assembly (Kutsukake et al., 1994). FlhB has two structural domains: the amino (N)-terminal domain (FlhB_TM_) and the carboxyl (C)-terminal domain (FlhB_C_). The amino acids linking these domains are critical for the function of FlhB. The deletion or mutation of these amino acids adversely affects T3SS-mediated transport (Fraser et al., 2003; Zarivach et al., 2008). The presence of Asn-Pro-Thr-His sequence within the C-terminal domain in *Salmonella* FlhB is required for FlhB function, and cleavage of the Asn-Pro bond is required for substrate switching process. Collectively, our results show that the transport protein FlhB is involved in the operation of the flagellar export in *L. monocytogenes*.

In addition to FlhB, FliM, and FliY play important roles in the flagellar system of Gram-negative bacteria. FliM is a C-ring component, which plays a role in flagellar assembly and CW/CCW switching of the direction of flagellar rotation (Onoue et al., 2016), in Gram-positive bacteria, its function and mechanism of operation have not been studied and confirmed. FliY is a flagellar rotor protein of the CheC phosphatase family, and it is localized in the flagellar switch complex, which contains the stator-coupling protein FliG and the target of CheY-P, FliM. Regions of FliM that mediate contacts within the rotor compose the phosphatase active sites in FliY. Despite the similarity between FliY and FliM, FliY does not bind FliG and thus is unlikely to be a substitute for FliM in the center of the switch complex (Sircar et al., 2013). It is clear that FliM and FliY are important flagellar-related proteins that are required for flagellar synthesis and export in *L. monocytogenes*.

**FIGURE 5:**
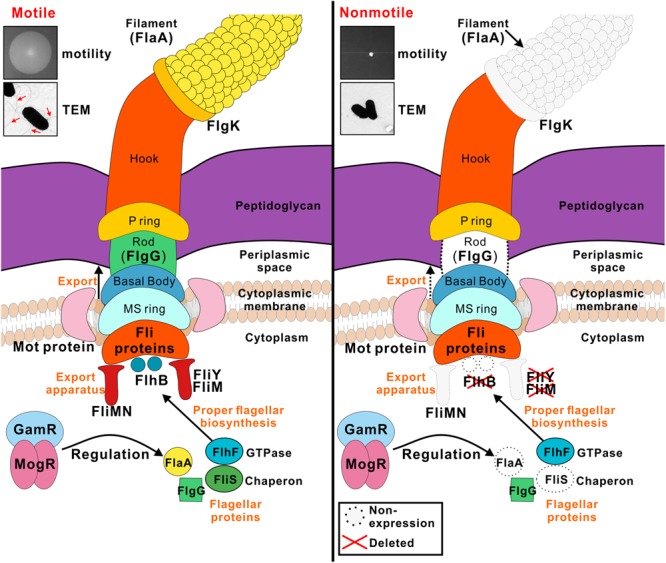
Model for the roles of FlhB, FliY, and FliM in flagellar formation and bacterial motility. FlhB as an export switch factor is responsible for transporting intracellular proteins to the extracellular, including FlgG and FlaA. Besides its role in the flagellar switch complex, the C-ring is also involved in the export process, FliM and FliY are components of the C ring, their defects may lead to a variety of specific substrate proteins that can not be transported. FliS chaperone binds to filament (FlaA) in the cytoplasm and efficiently transfers FlaA to a sorting platform of the flagellar export apparatus during the flagellar filament assembly. The *flhB, fliM*, and *fliY* mutant strains are incapable of synthesizing filament, thus causing the bacteria to lose power and non-motile. FlhF is a GTPase associated with flagellin localization and flagellar transcription is still subject to temperature-dependent regulation of MogR and GmaR strictly, all these three regulators are not disturbed by FlhB, FliM, and FliY.

In summation, FlhB, FliM, and FliY are involved in flagellum synthesis in *L. monocytogenes*, and they are required for motility and the secretion of other flagellar-related proteins. The expression and secretion of some other flagellar-related proteins, such as MogR, GmaR, the GTPase FlhF was not affected in the *flhB, fliM*, and *fliY* mutants, indicating that FlhB, FliM, and FliY partially affect flagellar function, and that there are other flagellar control mechanisms in *L. monocytogenes* (**Figure 5**). Studies of the functions of flagellar-related proteins in *L. monocytogenes* are in the preliminary stage, and we in the future aim to discover important effector proteins that are regulated by *flhB* and to determine whether there is a pathogen-associated T3SS in *L. monocytogenes* and, if so, whether its function is related closely to the flagellar T3SS.

## Materials and Methods

### Bacterial Strains, Plasmids, and Culture Conditions

The *L. monocytogenes* EGD-e strain was used as the wild-type strain. *Escherichia coli* DH5α was employed for cloning experiments and as the host strain for the plasmids pET30a(+) (Merck, Darmstadt, Germany), pIMK2, and pKSV7. The *E. coli* Rosetta (DE3) strain was used for protein expression. *L. monocytogenes* was cultured in brain-heart infusion (BHI) medium (Oxoid, Hampshire, England). The DH5α and Rosetta (DE3) strains were grown at 37°C in Luria–Bertani (LB) broth (Oxoid). Stock solutions of ampicillin (50 mg/ml), erythromycin (50 mg/ml), kanamycin (50 mg/ml), or chloramphenicol (50 mg/ml) was added to the media when necessary. All chemicals were obtained from Sangon Biotech (Shanghai, China), Merck, or Sigma–Aldrich (St. Louis, MO, United States) at the highest purity available (Cheng et al., 2015).

### Construction of Gene Deletion Mutants

The temperature-sensitive pKSV7 shuttle vector was used to create mutations in *L. monocytogenes* strain EGD-e. A homologous recombination strategy using the overlap extension (SOE) PCR procedure was used to construct in-frame deletions in the *flhB, fliM*, and *fliY* genes (Monk et al., 2008). DNA fragments containing homologous arms upstream and downstream of the genes of interest were obtained by PCR amplification of EGD-e genomic DNA using the SOE primer pairs listed in Supplementary Table S1. The resulting in-frame-deletion mutants were further verified by DNA sequencing (Sangon Biotech, Inc., Shanghai, China).

### Complementation of the Gene Deletion Mutants

To complement the *L. monocytogenes* Δ*flhB*, Δ*fliM*, and Δ*fliY* strains, we constructed three complemented strains using the integrative plasmid pIMK2, which harbors the constitutive *Listeria* promoter *P*_help_ as performed previously (Cheng et al., 2017b). We amplified the complete open reading frames of *flhB, fliM*, and *fliY* from EGD-e genomic DNA using the primer pairs listed in Supplementary Table S1 and inserted them downstream of *P*_help_ after digestion with appropriate restriction enzymes. Then, the resulting plasmids were electroporated into the corresponding *L. monocytogenes* Δ*flhB*, Δ*fliM*, and Δ*fliY* strains. The electroporated cells were plated on BHI agar containing kanamycin (50 μg/ml).

### Expression and Purification of Recombinant Proteins

The recombinant proteins used in this study were expressed as fusion proteins with an N-terminal histidine-tag using pET30a(+) as the expression vector (Cheng et al., 2017a). The *E. coli* Rosetta (DE3) strain was used as the expression host. The full-length open reading frames of the genes of interest from the EGD-e genome were amplified with the primer pairs listed in Supplementary Table S1, inserted into the pET30a(+) vector, and finally transformed into *Rosetta* competent cells. *Escherichia coli* cells harboring the recombinant plasmids were grown in 500 ml of LB medium supplemented with 50 μg/ml kanamycin at 37°C until the cultures reached an optical density at 600 nm (OD_600 nm_) of 0.6–0.8. Then, IPTG was added to a final concentration of 0.2 mM to induce protein expression for an additional 3 h, and the proteins purified using nickel-chelated affinity column chromatography.

### Preparation of Polyclonal Antibodies against the Recombinant Proteins

The purified recombinant proteins were used to raise polyclonal antibodies in New Zealand white rabbits according to a standard protocol as previously described (Cheng et al., 2015, 2017b). The rabbits were first immunized by sub-cutaneous injections of 500 μg of protein with an equal volume of Freund’s complete adjuvant (Sigma–Aldrich). After 2 weeks, the rabbits were boosted subcutaneously three times with 250 μg of protein in incomplete Freund’s adjuvant (Sigma–Aldrich) at 10-day intervals. The rabbits were bled approximately 10 days after the last injection.

### Growth Analysis in BHI Broth

The *L. monocytogenes* wild-type strain EGD-e, the Δ*flhB*, Δ*fliM*, and Δ*fliY* mutant strains, and the three complemented strains (CΔ*flhB*, CΔ*fliM*, and CΔ*fliY*) were grown overnight at 37°C in BHI broth with shaking. The cultures were collected by centrifugation at 5,000 × *g* at 4°C, washed once in phosphate-buffered saline (10 mM, pH 7.4), and the initial OD_600 nm_ was adjusted to 0.6. Then, the bacteria were diluted 1:100 into fresh BHI broth and incubated at 37°C and 30°C for 12 h. The OD_600 nm_ was measured at 1-h intervals.

### Motility Assay and TEM

The motility assay was performed on soft LB agar (0.25%) as described (Paxman et al., 2009; Cheng et al., 2017b). Specifically, *L. monocytogenes* strains were grown overnight in BHI medium, and the cultures were adjusted to an OD_600 nm_ of 0.20 (approximately 2 × 10^8^ CFU/ml). Bacterial samples (5 μL) were dropped onto the soft LB agar and incubated at 30°C or 37°C for 48 h to allow for growth. Motility was assessed by examining the outward migration of the bacteria through the agar from the point of inoculation. TEM experiments were performed as described previously (Dingle et al., 2011), in the Institute of Agrobiology and Environmental Sciences of Zhejiang University. Single *L. monocytogenes* colonies that were grown overnight at 30°C from BHI agar plates were suspended in 50 μL of monoethanolamine buffer (pH 10.0), and then 10 μL of the suspension was applied to carbon-coated copper grids and incubated for 2 min at room temperature. Excess liquid was subsequently wicked away using filter paper, and the bacteria were stained with 10 μL of 0.5% phosphotungstic acid (pH 7.0) for 10 s at room temperature. Excess stain was gently wicked away using filter paper, and the dried grids were examined using a Hitachi H-7650 transmission electron microscope (Hitachi High-Technologies Corporation, Tokyo, Japan).

### qRT-PCR Assay

The *L. monocytogenes* wild-type strain EGD-e and the Δ*flhB*, Δ*fliM*, and Δ*fliY* mutant strains were grown to stationary phase (OD_600 nm_ of 0.6) in BHI broth at 30°C without shaking. Total RNA was extracted using the Trizol reagent, and cDNA was synthesized with reverse transcriptase (TOYOBO, Osaka, Japan). Then, quantitative PCRs were performed by using the SYBR qPCR mix (TOYOBO) to measure the transcriptional levels of *mogR, fliN, fliP, fliQ, fliR, flhA, flhF, flgG, motA, motB, gmaR, flaA, fliNY, lmo0698, fliM, fliY, flgK, flgL, fliD, fliS, flgB, flgC, fliE, fliF, fliG, fliH*, and *fliI* using an iCycler iQ5 real-time PCR detection system (Bio-Rad, Hercules, CA, United States) with specific primer pairs. Relative transcription levels were quantified by the 2^-ΔΔC_T_^ method and are shown as relative fold changes (Livak and Schmittgen, 2001). The transcriptional analysis was repeated three times for each test condition.

### Changes in the Expression Levels of Flagellar-Associated Proteins in the Absence of *flhB, fliM, and fliY*

To further determine whether *flhB, fliM, and fliY* are involved in bacterial flagellar synthesis, western blotting was employed to analyze the changes in expression of major flagellar-associated factors. Bacterial overnight cultures of the *L. monocytogenes* wild-type EGD-e, the Δ*flhB*, Δ*fliM*, and Δ*fliY* mutant strains, and the three complemented strains (CΔ*flhB*, CΔ*fliM*, and CΔ*fliY*) were diluted into 100 ml of fresh BHI broth, and then the bacteria were grown to stationary phase. We used the fractionation procedure described by Lenz and Portnoy (2002), with minor modifications, to isolate secreted proteins. Briefly, the bacteria were pelleted by centrifugation at 13,000 × *g* for 20 min at 4°C, and the resulting culture supernatant was collected and then filtered through a 0.22-μm polyethersulfone membrane filter (Merck). Trichloroacetic acid was added to the supernatant to a final concentration of 10%. Proteins were trichloroacetic acid-precipitated on ice overnight and washed with ice-cold acetone (Cheng et al., 2017b). The washed precipitates of the supernatant proteins were re-suspended in sodium dodecyl sulfate–polyacrylamide gel electrophoresis (SDS-PAGE) sample buffer (5% SDS, 10% glycerol, and 50 mM Tris-HCl, pH 6.8). The samples were boiled for 5 min and stored at -20°C before electrophoresis. The method for total cell protein isolation was performed as described previously (Cheng et al., 2017b). The protein samples were separated using 12% SDS-PAGE and immunoblotted with α-FlgG, α-MogR, α-FlhF, α-FliM, α-FliY, α-FlaA, α-FliS, α-GmaR, and α-glyceraldehyde 3-phosphate dehydrogenase (GAPDH) antisera. GAPDH was used as an internal control.

### Statistical Analysis

Data were analyzed using a two-tailed homoscedastic Student’s *t*-test. Differences with *P* values < 0.05 were considered to be statistically significant.

## Ethics Statement

All animal care and use protocols were performed in accordance with the Regulations for the Administration of Affairs Concerning Experimental Animals approved by the State Council of People’s Republic of China. The protocol was approved by the Institutional Animal Care and Use Committee of Zhejiang A&F University (Permit Number: ZJAFU/IACUC_2011-10-25-02). All the *Listeria monocytogenes*-involved experiments in our study were conducted at Biosafety Level 2 (BSL-2) Laboratory.

## Author Contributions

CCh and HS conceived the study. CCh, HW, TM, JS, LJ, XH, YH, HY, and FL carried out the experiments. CCa, YY, LJ, and ZC analyzed the data. CCh, WF, and HS drafted the manuscript. All authors contributed to this study, prepared the final version of the manuscript, and read and approved the final manuscript.

## Conflict of Interest Statement

The authors declare that the research was conducted in the absence of any commercial or financial relationships that could be construed as a potential conflict of interest.
