# Supplementary material for: Flagellar Basal Body Structural Proteins FlhB, FliM, and FliY Are Required for Flagellar-Associated Protein Expression in Listeria monocytogenes
Source: Front Microbiol. 2018 Feb 13;9:208. doi: 10.3389/fmicb.2018.00208 (PMC5816908; doi:10.3389/fmicb.2018.00208)
Supplement: Supplementary file 1 [file Table_1.PDF]

Table S1. PCR Primers used in this study. Nucleotides introduced to create restriction enzyme sites are underlined. All primers were synthesized by GENEWIZ, Inc., Suzhou, China.

Primer name	Primer sequence(5'-3')	Product	Description
pSL309-up-fwd	CGGGATCCGACGCTTGGTTTTGCACTTG	522 bp	Used for construction of *flhB* null mutant
pSL309-up-rev	TGGCGCTGTTCTATAACTTTCCATGTTCGCTAGCT		
pSL309-dn-fwd	GAAAGTTATAGAACAGCGCCAAAGAAGCATT	578 bp	
pSL309-dn-rev	AACTGCAGAATCTTTAATTGATGTGGCACGC		
pSL309-a-front	ATACTTTTTTTGTAGTGATATTCTTGTCG		
pSL340-up-fwd	CGGGGTACCTACGTTCTCCAATGAAGACGGTTTAGT	553 bp	Used for construction of *fliM* null mutant
pSL340-up-rev	ATTGCTCCACTTTTTTTGTCACTCCCTAGCTCTCATGA		
pSL340-dn-fwd	GAGTGACAAAAAAAGTGGAGCAATTACTAGAGAAAAATATCAC	580 bp	
pSL340-dn-rev	GCGCTGCAGTTATCAACAGGAATGAGTTGCATTAAATT		
pSL340-a-front	AGTATTCTCACCAACTTCTGATGGCA		
pSL342-up-fwd	CGGGGTACCCTCACCAGAAATCGTTAATATCGAAACC	486 bp	Used for construction of *fliY* null mutant
pSL342-up-rev	TTCGCACATTTTTTCTACCTCTCCTGCTCTGTCCGC		
pSL342-dn-fwd	GAGAGGTAGAAAAAATGTGCGAAAACTTGGACTGATG	470 bp	
pSL342-dn-rev	CGGCTGCAGATTCTGCGTCATCCAACTCGGCT		
pSL342-a-front	TCGATTTAGCTTATATCATTTATATTCATGAATGTT		
pSL316-fwd	CATGCCATGGTTGGCGAAGGATAATAAAACGGAA	1066 bp	Used for complementation of the *flhB* deletion
pSL316-rev	CGCGGATCCTTAGAACTTGATTTTGTCTGCGTCCA		

pSL348-fwd	CATGCCATGGCTATGAGCGATAAATTAAGTCAAGAACAAATT	1014 bp	Used for complementation of the *fliM* deletion
pSL348-rev	CGCGGATCCCTACCTCTCCTGCTCTGTCCGC		
pSL349-fwd	CATGCCATGGCTGTGGAGCAATTACTAGAGAAAAATATCAC	1569 bp	Used for complementation of the *fliY* deletion
pSL349-rev	CGCGGATCCTCACCTTACTAACTCAGTCATTTGTATGCC		
pSL331-fwd	CGCCATATGAAGGGATTATACATTGGCGCAGC	792 bp	Used for recombinant FlgG expression in *E. coli*
pSL331-rev	CCGCTCGAGCTGACGAATTAATCCAGTTGCTTCTTT		
pSL332-fwd	CGCCATATGCCTAAATCAGAAATAAGAAAATTACTTCAAGAAAT	933 bp	Used for recombinant MogR expression in *E. coli*
pSL332-rev	CCGCTCGAGCATTTGTTTATAATTTTCTTTGAATACACCAAGT		
pSL333-fwd	CGCCATATGCGGCCGTTAATTTCGATTTGTATG	1926 bp	Used for recombinant GmaR expression in *E.coli*
pSL333-rev	CCGCTCGAGTCGATTGTTTGTAACAGTGTCTTGAAGC		
pSL339-fwd	CGGGGTACCATGGGAAAACATTTAGTAGAAAAGATGGAA	1242 bp	Used for recombinant FlhF expression in *E. coli*
pSL339-rev	CGCGGATCCTCATTCCACTACCTGCCTCCTATCC		
pSL341-fwd	CGGGGTACCATGAGCGATAAATTAAGTCAAGAACAAATTGA	1011 bp	Used for recombinant FliM expression in *E. coli*
pSL341-rev	CGCGGATCCCTACCTCTCCTGCTCTGTCCGCC		
pSL343-fwd	CGGGGTACCGAGCAATTACTAGAGAAAAATATCACGCAA	1563 bp	Used for recombinant FliY expression in *E. coli*
pSL343-rev	CGCGGATCCTCACCTTACTAACTCAGTCATTTGTATGCC		
pSL344-fwd	CGGGGTACCATGAAAGTAAATACTAATATCATTAGCTTGAAAACACA	882 bp	Used for recombinant FlaA expression in *E. coli*
pSL344-rev	CGCGGATCCTTAGCTGTTAATTAATTGAGTTAACATTTGCG		
pSL345-fwd	CGGGGTACCATGCAAGCTTGGAAACGATACACAC	405 bp	Used for recombinant FliS expression in *E. coli*
pSL345-rev	CGCGGATCCTCAGCCAAATGTGTCTTTGCTTGAT		
